# Gut proteome and microbiome alterations: Analysis of transverse colon samples from pathologically confirmed Alzheimer's disease patients

**DOI:** 10.1002/alz.71021

**Published:** 2026-01-13

**Authors:** Qiwen Cheng, Jennifer Nolz, Timothy Karr, Nicole Dorn, Benjamin Readhead, Rosa Krajmalnik‐Brown, Diego Mastroeni

**Affiliations:** ^1^ Biodesign Institute, Center for Health Through Microbiomes Arizona State University Tempe Arizona USA; ^2^ Biodesign Institute, Neurodegenerative Disease Research Institute Arizona State University Tempe Arizona USA; ^3^ Biodesign Institute, Center for Personalized Diagnostics & Proteomics Center Arizona State University Tempe Arizona USA; ^4^ School of Sustainable Engineering and the Built Environment Arizona State University Tempe Arizona USA

**Keywords:** Alzheimer's disease, amyloid beta, antimicrobial defense, complement, microbiome, oxidative stress, synapse, transverse colon

## Abstract

**INTRODUCTION:**

Alzheimer's disease (AD) has been regarded as a brain‐first disorder. Emerging evidence suggests that the gut may influence central nervous system pathology, but the mechanisms remain unclear.

**METHODS:**

We conducted a proteomic and microbial analysis of transverse colon samples from clinically and pathologically confirmed AD and control cases.

**RESULTS:**

In the AD gut samples, antimicrobial humoral response and oxidative stress response were downregulated, while catabolic processes and insulin signaling were upregulated. Several complement (e.g., C5) and synaptic (e.g., synaptophysin) proteins were downregulated. Amyloid beta 42 was detected at higher levels. *Christensenellaceae*, *Desulfovibrio*, and *Candida tropicalis* amplicon sequence variants were higher in abundance, while *Streptococcus*, *Lachnospiraceae*, *Blautia*, and *Nakaseomyces* were lower. In general, bacterial composition correlated with AD clinical variables such as plaque and tangle burden.

**DISCUSSION:**

These findings underscore the gut's possible involvement in AD pathogenesis and provide new insights into potential biomarkers and therapeutic targets.

**Highlights:**

- This study provides the first in‐depth analysis of the proteome and microbiome in AD transverse colon tissues.
- Multiple immune and oxidative stress response pathways were downregulated in AD, while metabolic pathways were upregulated.
- Synaptic protein, complement protein, and Aβ42 levels were significantly different between AD and controls.
- Transverse colon microbial composition was associated with AD clinical variables.

## 1 BACKGROUND

Alzheimer's disease (AD), the leading cause of dementia, is marked by progressive cognitive decline and neuropathological features, including extracellular amyloid beta (Aβ) plaques and intracellular neurofibrillary tangles composed of hyperphosphorylated tau protein.^1^ Although traditionally regarded as a brain‐centric disorder, increasing evidence highlights the significant role of peripheral systems, particularly the gut–brain axis, in modulating disease progression and symptomatology.^2^ The gut–brain axis is a bidirectional communication network linking the gastrointestinal (GI) system and the central nervous system (CNS).^3^ This network is integral to regulating immune responses, inflammation, and metabolic function, all of which are implicated in AD pathology.^4^, ^5^

The gut has gained significant interest due to its dual role as both a potential source and regulator of pathogenic mechanisms, with emerging research underscoring the association between the gut–brain axis and AD‐related pathology. For example, Aβ, a hallmark protein of AD, has been detected in non‐CNS tissues, including the GI tract, raising the intriguing possibility that the gut may serve as a reservoir or production site for this protein.^1^, ^6^, ^7^ This insight complements the concept of the gut–brain axis as a conduit through which peripheral changes might influence CNS pathology, linking systemic alterations to neurodegeneration.

The gut microbiome—a diverse community of microorganisms residing in the GI tract—has become a key player in neurodegeneration.^2^, ^7^, ^8^, ^9^, ^10^ It influences brain function through neuroactive metabolite production, immune system modulation, and gut permeability regulation. For instance, short‐chain fatty acids (SCFAs) like acetate, propionate, and butyrate, produced via anaerobic fermentation of non‐digestible carbohydrates, exhibit neuromodulatory effects by interacting with enteroendocrine cells in the GI tract.^11^ SCFAs can also regulate tau phosphorylation, a pathological hallmark of AD.^1^, ^12^ It has been demonstrated that propionate and butyrate reduce tau phosphorylation by modulating activity in enteroendocrine cells, which connect directly to enteric neurons and glial cells.^12^ These findings align with evidence that hyperphosphorylated tau drives AD pathology.^13^ Furthermore, enteroendocrine cells have been shown to express α‐synuclein (α‐syn), a presynaptic neuronal protein associated with neurodegeneration, further implicating the gut as a potential contributor to AD pathology.^12^

Dysbiosis, an imbalance in the gut microbiome, is frequently observed in AD patients. Studies report increased abundances of proinflammatory bacteria such as proteobacteria and reduced levels of anti‐inflammatory taxa like *Firmicutes*.^14^ This microbial imbalance contributes to systemic inflammation through mechanisms such as increased gut permeability, also known as “leaky gut.” A compromised intestinal barrier allows pathogens, inflammatory metabolites, and bacterial endotoxins such as lipopolysaccharide (LPS) to enter the bloodstream, triggering immune responses and chronic inflammation.^15^ This inflammation exacerbates neuroinflammation, promoting the activation of microglia—the brain's resident immune cells—which are implicated in driving the neurodegenerative processes characteristic of AD.^16^

Despite the burgeoning interest in gut involvement, most existing studies have focused on fecal samples, which provide a limited view of the gut environment. Consequently, critical gaps remain regarding the microbiome composition and its functional dynamics within colonic tissues of AD patients. In this study, we conducted an integrated proteomic and microbial analysis of the transverse colon in clinically and pathologically confirmed AD and control subjects. Using liquid chromatography tandem mass spectrometry (LC‐MS/MS) alongside 16S rRNA gene amplicon sequencing, we aimed to characterize gut proteome and microbiome differences associated with AD. Our findings shed light on the interplay among gut dysbiosis, altered protein expression, and peripheral inflammation, advancing our understanding of how the gut–brain axis may contribute to AD pathology.

## 2 MATERIALS AND METHODS

### 2.1 Study participants and sample collection

Transverse colon (TVC) samples were obtained from 26 AD patients and 28 age‐matched control participants in the brain and body donation program Alzheimer's disease center at Banner Sun Health Research Institute (BSHRI; Table 1 and Table S1 in supporting information [individual sample detail]), all of whom were clinically and pathologically confirmed. All participants self‐identified as of White/European ancestry. Transverse colon samples were obtained from the same individuals who provided brain tissues, ensuring matched clinical data and identical *post mortem* intervals (PMIs). Written informed consent for autopsy was obtained in compliance with institutional guidelines of BSHRI. The BSHRI Institutional Review Board approved this study including recruitment, enrollment, and autopsy procedures. Individuals involved in the study and their respective next of kin consented to brain and body autopsy for the purpose of research analysis as participants in the brain and body donation program Alzheimer's Disease Center. Human transverse colon samples used in this study were from routine existing autopsies, which qualify for exemption by the National Institutes of Health guidelines. In addition, samples were de‐identified and analyzed anonymously.

**TABLE 1:** Participant demographics and clinical characteristics. Dash (—) indicates that no statistical test was performed due to lack of variation. The MWU test was performed for continuous variables instead of *t* test (*t*) when data violated the assumption of normality.

Variable	AD	Control	*P* value (method)
Sample size	26	28	NA
**Demographic information**			
Race	All White	All White	—
Sex, male/female	18/8	16/12	0.52 (*χ* ^2^)
Expired age (in years), M (SD)	82 (8.4)	81 (9.8)	0.80 (*t*)
**Clinical variable**			
*Post mortem* interval (in hours), M (SD)	3.3 (0.85)	3.1 (0.91)	0.58 (MWU)
Mini‐Mental State Examination, M (SD)	12 (8.0)	28 (1.8)	6.0E‐10 (MWU)
Apolipoprotein E ε4 gene carrier, *n* (%)	14 (54%)	4 (14%)	0.0052 (χ^2^)
Total count of plaques, M (SD)	14 (1.3)	2.4 (4.3)	4.3E‐10 (MWU)
Total count of neurofibrillary tangles, M (SD)	14 (1.9)	3.1 (2.0)	2.0E‐10 (MWU)
Duration of dementia (in years), M (SD)	9.2 (5.3)	NA	1.2E‐11 (MWU)
TVC amyloid beta 42 levels (in pg/mL), M (SD)	66 (21)	24 (15)	6.0E‐08 (MWU)

Abbreviations: AD, Alzheimer's disease; M, mean; MWU, Mann–Whitney *U* test; NA, not applicable; SD, standard deviation; TVC, transverse; χ^2^, Chi‐squared test.

### 2.2 Tissue selection and quality control

In this study, we focused on the transverse colon due to its unique anatomical and functional characteristics that may make it particularly relevant to AD pathogenesis. Recent quantitative mapping of the human enteric nervous system (ENS) revealed that the transverse colon harbors the highest density of myenteric neurons among colonic regions, surpassing both proximal and distal sites, and thus may offer enhanced sensitivity for detecting gut–neural alterations in AD.^17^ Its proximity to the middle colic artery, a branch of the superior mesenteric vasculature, may also increase its exposure to systemically circulating metabolites such as Aβ. Additionally, the transverse colon represents a microbial and metabolic transition zone, exhibiting distinct microbial communities shaped by intermediate nutrient availability, pH, and motility, features that differentiate it from both fecal content and more distal segments.^18^, ^19^ Together, these anatomical, vascular, and microbial features make the transverse colon a compelling site for probing gut contributions to AD pathophysiology.

**RESEARCH IN CONTEXT**

1. **Systematic review**: The authors reviewed the relevant literature using PubMed. Existing studies indicate that the gut may play a role in influencing Alzheimer's Disease (AD) pathology; however, most research has focused on fecal samples, not colonic tissues.
2. **Interpretation**: Our findings suggest that in colonic tissues the complement and synaptic dysfunction, along with microbial dysbiosis, were associated with AD pathology. The identification of amyloid β42 in gut tissues adds a novel dimension to the systemic nature of AD.
3. **Future directions**: Longitudinal studies and interventional trials that account for confounding factors are crucial to clarify the gut‐AD relationship. Comparing gut tissue with fecal matter is essential for non‐invasive tracking of synaptic dysfunction and microbiome dysbiosis, which facilitates biomarker discovery for early diagnosis and potential therapeutic interventions.

Colon tissues were harvested at autopsy and immediately flash frozen on dry ice. Samples were stored at −80°C until processing. Prior to the proteomic analysis, tissue samples were assessed for gross integrity and suitability via macroscopic evaluation of mucosal architecture, absence of autolysis, and preservation of tissue color and texture by a pathologist. In addition, total protein yield was quantified using the bicinchoninic acid (BCA) assay (Thermo Fisher), ensuring a sufficient concentration and integrity for the downstream proteomic analysis. RNA integrity was assessed on all samples using a bioanalyzer as a secondary quality check. All samples included in the study had RNA integrity number values of 8.2 to 8.7 (mean  =  8.4), indicating high‐quality preservation suitable for downstream analysis.

### 2.3 Clinical and pathological assessment

The control subjects had no memory complaints or history of memory complaints, cognitive function within 1.5 standard deviations of the age‐ and education‐adjusted norms, Clinical Dementia Rating (CDR) score of zero, Mini‐Mental State Examination (MMSE) score of 25 to 30 (inclusive), and Braak staging < II. For AD cases, the following criteria were applied to ensure a clear distinction from the control subjects: presence of persistent and escalating clinical memory complaints, cognitive function exhibiting a decline of more than 1.5 standard deviations from age‐ and education‐adjusted norms, CDR score > 9, MMSE score < 12, and Braak staging equal to or exceeding Braak stage IV, indicative of the pathological progression associated with AD. *Post mortem* tissue samples were collected with a standardized PMI to minimize degradation < 4.3 hours.

### 2.4 Sample preparation, LC‐MS/MS, and label‐free quantification

All tissue sample preparation and LC‐MS/MS analyses were performed at the Biosciences Mass Spectrometry Core Facility (https://cores.research.asu.edu/massspec/) at Arizona State University. Briefly, tissue samples were solubilized and reduced in 25 µL of 5% sodium dodecyl sulfate (SDS)/50 mM tetraethylammonium bromide (TEAB) containing 50 mM dithiothreitol and incubated for 15 minutes at 95°C. Samples were then spun again at 15,000 rpm (20,000 xg) for 15 minutes at 20°C and visually inspected to ensure no visible pellets were present. Solubilized proteins were then removed and quantified using EZQ Protein Quantitation Kit (Thermo Fisher), and 15 µg of total protein alkylated for 30 minutes in the dark at room temperature by addition of 40 mM final concentration of freshly prepared iodoacetamide (Pierce). Peptides were prepared using the S‐trap Micro Columns (Protifi) following the manufacturer's S‐trap Micro High Recovery Protocol. Samples (≈ 30 µL) were acidified to 1.2% phosphoric acid by addition of a stock 12% phosphoric acid solution. Proteins were digested by addition of 2 µL of a 1 mg/mL solution of porcine (MS sequencing grade modified trypsin, Promega) and layered onto the S‐trap column containing 180 µL of 90% methanol/100 mM TEAB. Samples were briefly spun to remove excess buffer and washed four times with S‐trap buffer. An additional 0.5 µg of trypsin and 25 µL of 50 mM TEAB was added to the top of each column and incubated for 1 hour at 47°C. Samples were eluted off the S‐trap columns using three elution buffers: 50 mM TEAB, 0.2% formic acid in water, and 50% acetonitrile/50% water + 0.2% formic acid. Samples were dried down via a SpeedVac vacuum concentrator (Thermo Scientific) and resuspended in 30 µL of 0.1% formic acid.

All data‐dependent mass spectra were collected in positive mode using direct injection into an Orbitrap Fusion Lumos mass spectrometer (Thermo Scientific) coupled with an UltiMate 3000 Ultra‐High‐Performance Liquid Chromatograph (Thermo Scientific). One microliter of peptides was fractionated using an Easy‐Spray LC column (50 cm × 75 µm ID, PepMap C18, 2 µm particles, 100 Å pore size, Thermo Scientific). Electrospray potential was set to 1.6 kV and the ion transfer tube temperature to 300°C. The mass spectra were collected using the “universal” method optimized for peptide analysis provided by Thermo Scientific. Full MS scans (375–1500 *m*/*z* range) were acquired in profile mode with the Orbitrap set to a resolution of 120,000 (at 200 m/z), cycle time set to 3 seconds, and mass range set to “normal.” The RF lens was set to 30% and the AGC set to “standard.” Maximum ion accumulation time was set to “auto.” Monoisotopic peak determination was set to “peptide” and included charge states 2 to 7. Dynamic exclusion was set to 60 seconds with a mass tolerance of 10 ppm and the intensity threshold set to 5.0e3. MS/MS spectra were acquired in a centroid mode using quadrupole isolation window set to 1.6 m/z. Collision‐induced fragmentation energy was set to 35% with an activation time of 10 ms. Peptides were eluted using a 150‐min nanoLC gradient at a flow rate of 0.250 µL/min with increasing acetonitrile in water: 0–10 min, 2–10%; 10–110 min, 10–32%; 110–120 min, 32–35%; 120–125 min, 35–42%; 125–130 min, 42–98%; 130–140 min, 98%; and 140–150 min, 98–2%.

Label‐free quantification (LFQ) intensity values obtained from the Minora Feature Detector were log2‐transformed and median normalized across samples to correct for differences in total protein loading in Proteome Discoverer 2.4. Proteins were retained for analysis if they were quantified in at least 50% of samples in one experimental group. Missing values were handled according to this mechanism: values considered missing at random were imputed using k‐nearest neighbors (*k* = 5), while values missing not at random (typically absent in one condition) were imputed from a left‐censored normal distribution (width = 0.3, downshift = 1.8 standard deviations). Differentially abundant proteins between AD and control samples were identified using the limma package (version 3.60.6), applying linear models with empirical Bayes moderation. Diagnostic group (AD vs. controls) was the primary factor of interest, with sex, PMI, and age at death (ExpiredAge) included as compounding variables. *P* values for proteins were adjusted for multiple comparisons using the Benjamini–Hochberg false discovery rate (FDR) method. Quality control included inspection of principal component analysis plots, relative log expression plots, and assessment of mean–variance trends. Technical reproducibility was evaluated using median coefficients of variation across replicates.

Proteins with an FDR < 0.05 were retained for pathway enrichment analysis against the Gene Ontology, Kyoto Encyclopedia of Genes and Genomes, and Reactome databases using gene set enrichment analysis. Cytoscape (Cytoscape.org) was used for network analysis, integrating proteomic data with reference single‐cell RNA sequencing (scRNA‐seq) atlases. Transverse colon proteomics were mapped to a human colon scRNA‐seq reference, enabling inference of cell type–specific protein expression. This approach facilitated spatial reconstruction of protein localization within tissue, highlighting the distribution of proteins linked to AD pathology and gut dysbiosis.

### 2.5 Microbiome analysis

DNA was extracted from 54 transverse colon samples using the DNeasy PowerSoil Pro Kit (QIAGEN). The V4 region of the bacterial 16S rRNA gene was amplified with the primers 515F (5′‐GTGCCAGCMGCCGCGGTAA) and 806R (5′‐GGACTACHVGGGTWTCTAAT), and sequenced using the Illumina MiSeq platform (2 × 250). QIIME 2 (version 2023.5)^20^ was used for sequence quality control, feature table and representative sequence construction, and phylogenetic tree generation. The DADA2 plugin^21^ was used to filter and merge the forward and reverse sequences. Sequences were then mapped to the Silva 138 SSURef NR99 database for the microbiome composition analysis.^22^, ^23^, ^24^ A classifier was trained with the forward and reverse primers used in this study.

The fungal ITS region was amplified with the primers ITS1F (5′‐CTTGGTCATTTAGAGGAAGTAA) and ITS2R (5′‐GCTGCGTTCTTCATCGATGC), and sequenced using the Illumina MiSeq platform (2 × 300). Only forward sequences were kept for downstream analyses as the reverse sequences were of low quality. In QIIME 2 (version 2023.5),^20^ the cutadapt plugin^25^ was used to remove adaptor sequences, followed by the DADA2 plugin^21^ for quality control and feature data construction. We observed a bimodal distribution in the number of fungal sequences after DADA2 processing (≥ 12,801 or ≤ 2379), and we decided to exclude samples with < 2379 sequences which did not contain enough DNA for sequencing. Sequences were mapped to the UNITE database (version 10, 99% sequence similarity) for fungal composition analysis, using a classifier trained with the ITS1F and ITS2R primers.^22^, ^23^, ^24^

The feature table, taxonomy, and rooted tree files from QIIME2 and metadata were imported into R^26^ using the qiime2R package (version 0.99.6),^27^ and then rarified using the phyloseq package (version 1.48.0)^28^ for diversity analyses. The rarefaction depth was 19,886 for 16S rRNA gene sequences and 12,801 for fungal sequences, to match the smallest library sizes for 16S rRNA gene and fungal ITS data. Alpha diversity metrics for 16S rRNA gene data, including Observed Amplicon Sequence Variants (Observed ASVs), Shannon's Diversity, and Faith's Phylogenetic Diversity (Faith's PD), were calculated using the R packages microbiome (version 1.26.0)^29^ and picante (version 1.8.2),^30^ and visualized using the R ggplot2 package (version 3.5.1).^31^ The Bray–Curtis dissimilarity was calculated using the R rbiom package (version 1.0.3),^32^ and visualized using the R graphics package (version 4.4.1)^26^ for 16S rRNA gene data and ggplot2 package^31^ for fungal ITS data.

All statistical analyses for microbiome and mycobiome were conducted in R. The differentially abundant bacterial proteins were determined as described in section 2.4. The “kruskal.test” function in the R stats package (version 4.4.1)^26^ was used to compare alpha diversity metrics between AD and control samples. Permutational analyses of variance were conducted with the “adonis2” function (permutations = 9999, other settings as default) in the R vegan package (version 2.6‐8)^33^ to examine associations between Bray–Curtis dissimilarity and each AD clinical variable. The “ancombc” function in the R package ANCOMBC (version 2.6.0)^34^ was used to identify differentially abundant ASVs between neurological diagnostic groups (AD vs. controls). Moreover, correlations between ASV abundances and each clinical variable were analyzed. The variables include the MMSE score, the total number of plaques (PlaqueTotal) and neurofibrillary tangles (TangleTotal) in the subject's brain, the number of years the subject had suffered from dementia (DementiaYears), Aβ42 levels (Aβ42), and apolipoprotein E (*APOE*) ε4 gene carrier status (treated as a categorical variable). All the models were adjusted for the confounding variables sex, PMI, and ExpiredAge. *P* values were corrected using the default Holm method. Only ASVs with a prevalence of > 20% and a detection threshold of 1 were included in the differentially abundance analyses. For all the tests, a *P* value of < 0.05 was considered statistically significant.

The raw 16S and ITS sequence data were deposited in the National Center for Biotechnology Information (NCBI) Sequence Read Archive (SRA) database (BioProject accession number: PRJNA1234023).

### 2.6 Aβ detection

#### 2.6.1 Western blot analysis of Aβ (6E10) in transverse colon tissue

AD transverse colon samples were homogenized for western blotting to detect Aβ species using the monoclonal antibody 6E10. Approximately 30 to 50 mg of frozen tissues were homogenized in ice‐cold RIPA buffer (50 mM Tris‐HCl pH 7.4, 150 mM NaCl, 1% NP‐40, 0.5% sodium deoxycholate, and 0.1% SDS) supplemented with a protease inhibitor cocktail (AEBSF/aprotinin/E64/leupeptin; Merck/MilliporeSigma, P8340). Homogenization was performed using a Dounce homogenizer, followed by incubation on ice for 30 minutes with intermittent mixing. Lysates were centrifuged at 16,000 × g for 20 minutes at 4°C, and the clarified supernatant was collected. Protein concentration was determined using the BCA assay (Pierce).

Equal amounts of protein (30 µg per lane) were mixed with 4 × Laemmli buffer containing β‐mercaptoethanol and heated at 95°C for 5 minutes. Samples were loaded onto gradient 10% to 20% Tris‐Tricine gels. Electrophoresis was performed under reducing conditions, followed by transfer onto polyvinylidene difluoride membranes (0.2 µm pore) using wet transfer at 100 V for 60 to 75 minutes.

Membranes were blocked for 1 hour at room temperature in 5% bovine serum albumin (BSA; Fraction V, protease‐free; Merck/MilliporeSigma, A7030) prepared in TBST (Tris‐buffered saline [TBS] + 0.1% Tween 20). Blots were incubated overnight at 4°C with monoclonal antiAβ antibody 6E10 (1:1000 dilution) in 2% BSA/TBST. After washes, membranes were incubated with horseradish peroxidase–conjugated antimouse immunoglobulin G (1:5000 in TBST) for 1 hour at room temperature. Detection was performed using chemiluminescent substrate and imaged using a digital imaging system.

#### 2.6.2 Quantification of Aβ42 by enzyme‐linked immunosorbent assay

For quantification of Aβ42, ≈ 100 mg of transverse colon tissues were homogenized in 500 µL of an extraction buffer consisting of 5 M guanidine‐HCl and 50 mM Tris pH 8.0 (prepared by dissolving 14.33 g guanidine‐HCl in ≈ 20 mL ultrapure water, adding 1.50 mL of 1 M Tris pH 8.0, and adjusting the final volume to 30.0 mL). Homogenization was performed using a Dounce homogenizer, and samples were extracted for 3 hours at room temperature on a rotating stand. Extracts were diluted 1:100 by mixing 10 µL of homogenate with 990 µL of Working Diluent, which was prepared fresh by supplementing the kit's Standard Diluent Buffer with 1× protease inhibitor cocktail (P8340). Diluted samples were centrifuged at 16,000 × g for 20 minutes at 4°C, and the clarified supernatant was collected and kept cold prior to assay. Quantification of Aβ42 was performed using the Human β‐Amyloid 1‐42 enzyme‐linked immunosorbent assay (ELISA) Kit (Thermo Fisher Scientific, KHB3441). To ensure accurate matrix matching, all standards and samples were diluted in Standard Diluent M, consisting of Working Diluent supplemented with 0.05 M guanidine‐HCl (prepared by adding 100 µL of 5 M guanidine‐HCl to 9.90 mL Working Diluent). The lyophilized Aβ42 standard was reconstituted to 2000 pg/mL in deionized water, allowed to equilibrate for 10 minutes, and serially diluted 1:2 in Standard Diluent M to generate standards of 1,000, 500, 250, 125, 62.5, 31.25, and 15.63 pg/mL, with Standard Diluent M alone serving as the 0 pg/mL blank. The assay was conducted according to the manufacturer's protocol, with 50 µL of each sample loaded per well in duplicate. Absorbance was measured at 450 nm using a microplate reader, and Aβ42 concentrations were calculated from the standard curve. Comparisons between AD (*n* = 26) and control (*n* = 28) groups were performed using the Mann–Whitney *U* test due to non‐normal data distribution, with *P* < 0.05 considered statistically significant.

## 3 RESULTS

### 3.1 Pathway network analysis of biological processes in AD revealed downregulation of important biochemical processes

**FIGURE 1:**
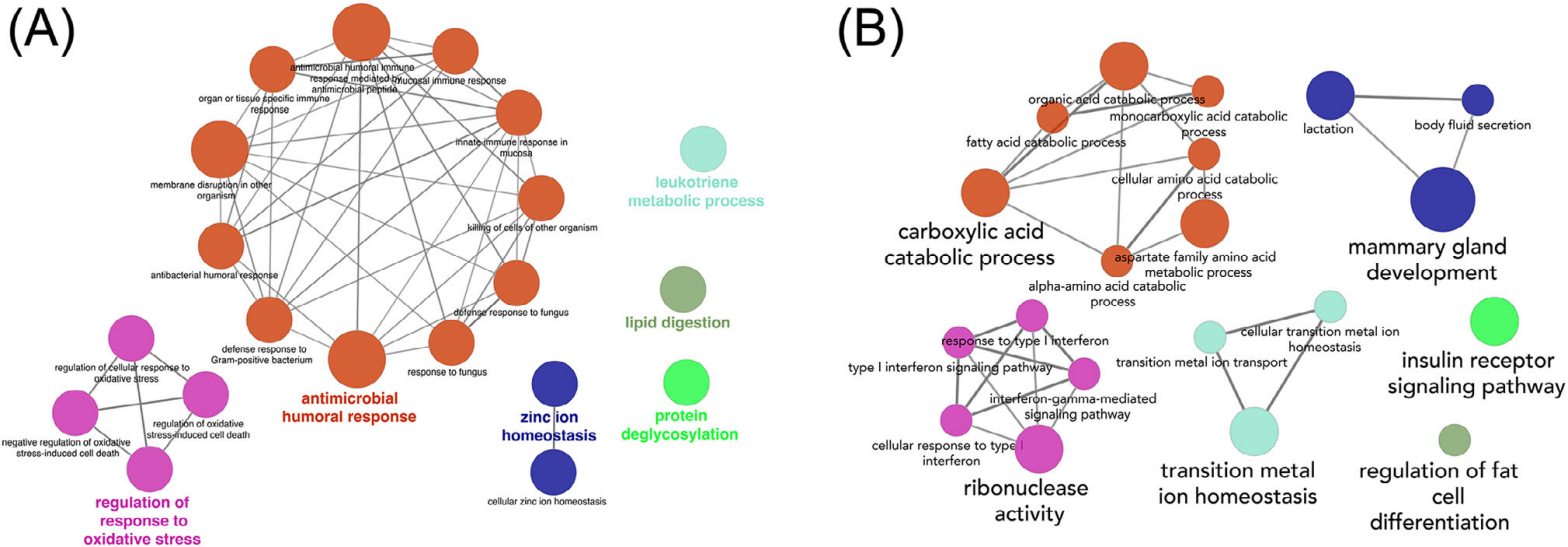
Pathway network analysis of altered biological processes in the transverse colon of Alzheimer's disease (AD) patients compared to controls. Each node represents a specific biological process, with the size of the node corresponding to the significance of that process. The connecting lines between nodes indicate shared genes or interactions between these biological processes. The color‐coding groups relate processes together. A, Downregulated biological processes in AD. Orange nodes indicate antimicrobial and immune response pathways, purple nodes indicate oxidative stress response pathways, and nodes in other colors indicate processes such as lipid digestion, zinc ion homeostasis, and protein deglycosylation. B, Upregulated biological processes in AD. Major biological processes are highlighted, including carboxylic acid catabolic processes, mammary gland development, and insulin receptor signaling pathway

Pathway enrichment analysis of the proteomic data revealed downregulation of several key biological processes in the transverse colon of AD patients compared to controls (Figure 1A). The most prominently downregulated pathways were related to antimicrobial humoral response, which encompasses the body's defense against pathogens, including bacteria and fungi.^35^ Notably, processes such as antibacterial humoral response, organ‐ or tissue‐specific immune response, and membrane disruption in other organisms were significantly decreased in AD patients. Additionally, pathways involved in the regulation of response to oxidative stress were significantly diminished in AD samples. Processes such as regulation of oxidative stress–induced cell death and regulation of cellular response to oxidative stress were markedly lower in AD patients. These findings are consistent with previous reports linking oxidative stress to neurodegenerative processes in the brain, further highlighting the gut's potential contribution to systemic inflammation in AD.^36^

In contrast to the downregulation of immune and oxidative stress responses, several metabolic pathways were significantly upregulated in the transverse colon of AD patients (Figure 1B). Key upregulated processes included the carboxylic acid catabolic process, fatty acid catabolic process, and amino acid metabolic process, all of which are integral to the breakdown and use of energy‐rich molecules. Notably, the insulin receptor signaling pathway was significantly upregulated in AD, suggesting that insulin signaling pathways may also be affected in peripheral tissues. Furthermore, pathways related to transition metal ion homeostasis, particularly zinc ion transport, were upregulated in AD samples.

### 3.2 Complement proteins altered in the transverse colon of AD patients

**FIGURE 2:**
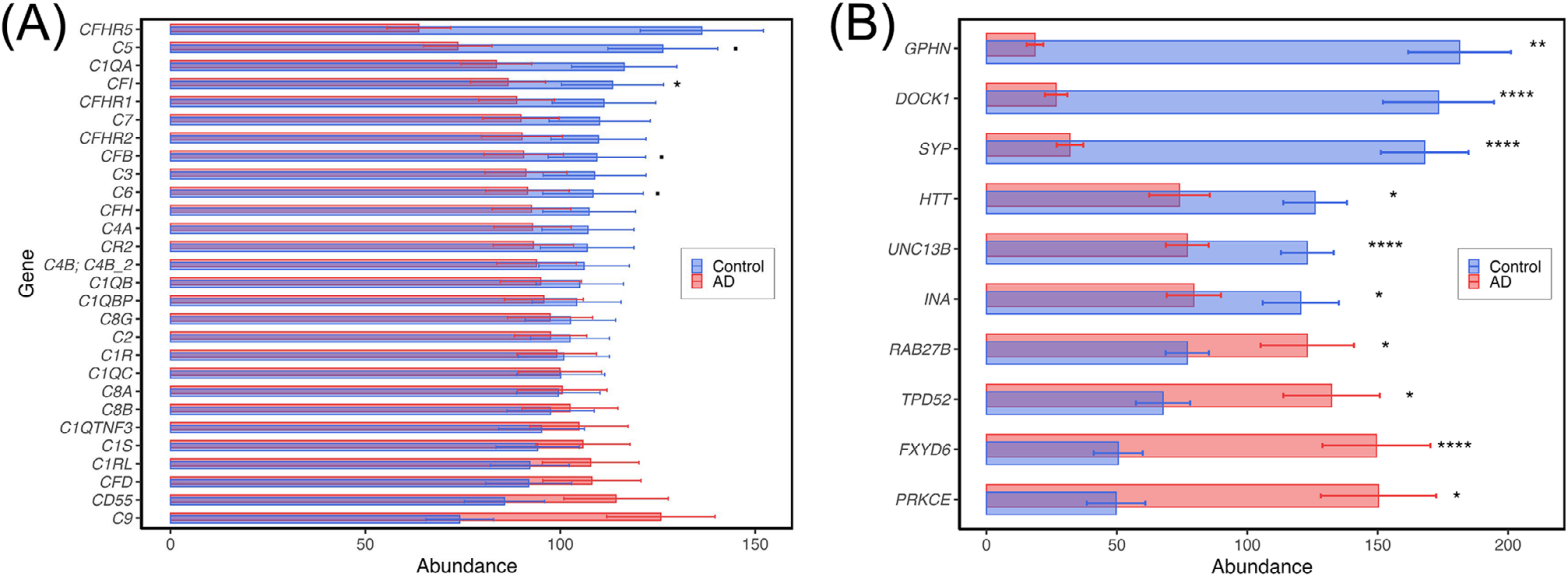
Bar graph depicting the grouped abundance of key complement and synaptic proteins (denoted by gene symbols) in the transverse colon of Alzheimer's disease (AD) patients compared to controls. A, Grouped abundance of complement proteins. B, Grouped abundance of synaptic proteins. The grouped abundance reflects normalized intensity values from label‐free quantification, aggregated across all samples within each group (26 AD and 28 control cases). Error bars denote standard error. •: *P* = 0.05; *: *P* < 0.05; **: *P* < 0.01; ****: *P* < 0.0001 (all Benjamini–Hochberg corrected)

Quantitative proteomic profiling revealed altered expression of multiple complement proteins in the transverse colon of AD patients compared to age‐matched controls (Figure 2A). Proteins with the strongest evidence for downregulation included CFI (AD: 87 ± 9.6 vs. controls: 113 ± 13, adjusted *P* = 0.013), C5 (74 ± 8.8 vs. 126 ± 14, adjusted *P* = 0.050), C6 (92 ± 11 vs. 108 ± 13, adjusted *P* = 0.051), and CFB (91 ± 10 vs. 109 ± 13, adjusted *P* = 0.050). These proteins are integral to classical, alternative, and terminal complement pathways.

Although not all reached statistical significance, broader downward trends were observed across additional complement components, including C1QA, C1QB, C3, C4A, C4B, and factor H‐related proteins CFHR1, CFHR2, and CFHR5. In contrast, proteins such as C8B, C1RL, and C1S showed mild increases in AD tissue, but with adjusted *P* values > 0.05. Notably, C9 (126 ± 14 vs. 74 ± 8.7) was elevated in AD, although this difference was not statistically significant (adjusted *P* = 0.98).

Together, these findings indicate selective suppression of key complement proteins in the AD colon, particularly those involved in activation cascades and immune regulation.

### 3.3 Peripheral synaptic proteins were downregulated in the transverse colon of AD patients

Proteomic analysis identified significant changes in synaptic proteins in the transverse colon of AD patients compared to age‐matched controls (Figure 2B). SYP and UNC13B, key components of the presynaptic machinery,^37^, ^38^ were significantly reduced in AD, showing 5.3‐fold and 1.6‐fold decreases, respectively (adjusted *P*s = 5.1E‐16). Other significantly downregulated proteins included DOCK1, GPHN, HTT, and INA (adjusted *P*s < 0.05).

Conversely, several synaptic proteins were elevated in AD tissues (Figure 2B). These included FXYD6, PRKCE, TPD52, and RAB27B, all of which showed statistically significant increases in abundance compared to controls (adjusted *P*s < 0.05).

### 3.4 Multiple Aβ‐immunoreactive species and elevated Aβ42 levels detected in the transverse colon of AD patients

**FIGURE 3:**
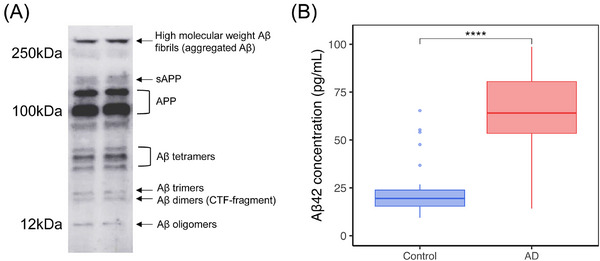
Aβ presence in the transverse colon of AD patients and controls. A, Western blot analysis showing the presence of various forms of Aβ in an AD patient, including high molecular weight fibrils, oligomers, and precursor forms like APP and sAPP. The detection of these species in the gut suggests that Aβ aggregation was not confined to the brain but also occurred in peripheral tissues, such as the gastrointestinal tract. B, Boxplot analysis showing elevated Aβ42 concentration in AD patients (*n* = 26) compared to controls (*n* = 28). The elevated levels of Aβ42 in the gut indicate that the gastrointestinal tract might actively participate in the disease process. ****: *P* < 0.0001. Aβ, amyloid beta; AD, Alzheimer's disease; APP, amyloid precursor protein; sAPP, soluble amyloid precursor protein

Western blot analysis using the 6E10 antibody demonstrated the presence of multiple Aβ species in transverse colon tissue, including monomers, oligomers, and APP fragments, confirming that Aβ is detectable in the human AD colon (Figure 3A). Quantitative ELISA further revealed that AD patients exhibited significantly elevated levels of Aβ42, with a mean concentration of 66 pg/mL in AD compared to 24 pg/mL in controls (*P* = 6.0E‐08; Table 1 and Figure 3B). These findings show multiple Aβ‐immunoreactive species and increased peripheral Aβ42 burden in the colon of individuals with AD.

### 3.5 Gut microbiome composition was altered in the transverse colon of AD patients

**FIGURE 4:**
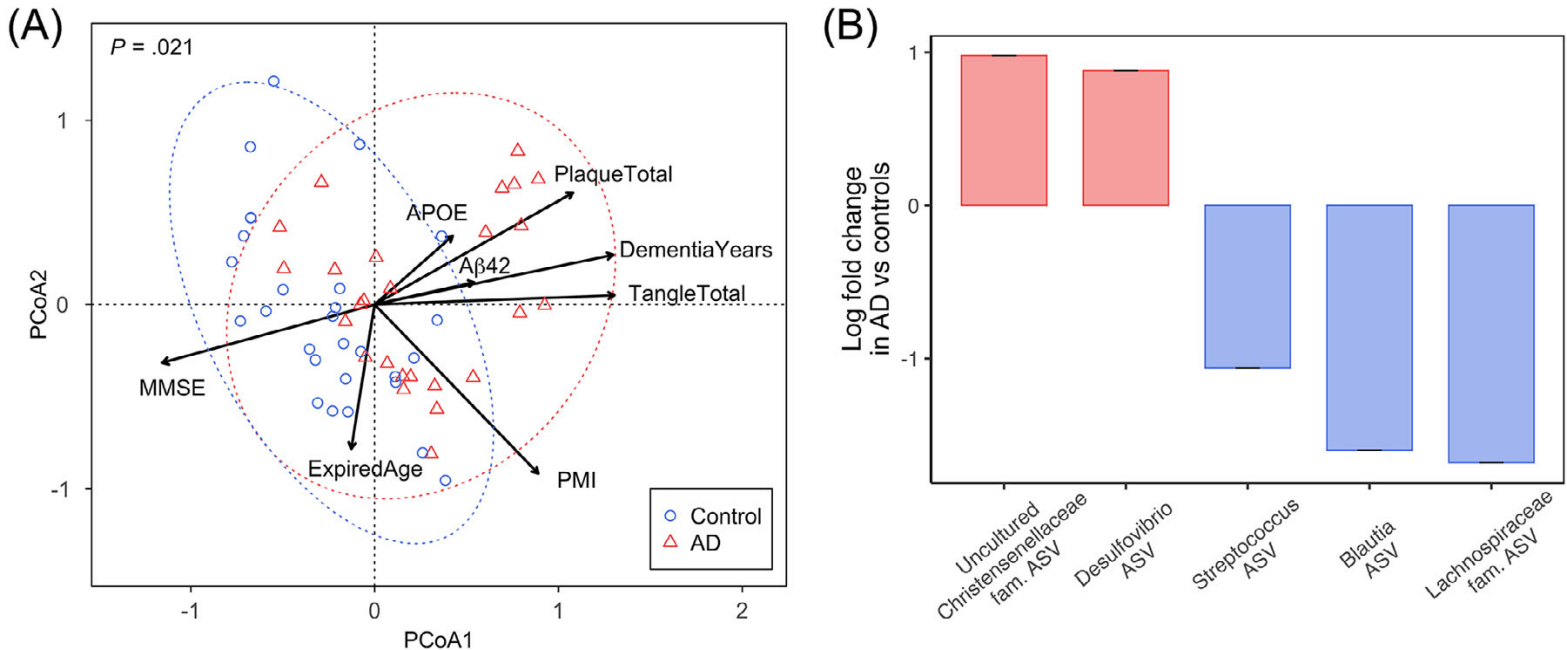
Comparison of bacterial composition in the transverse colon between AD patients and controls. A, Distance‐based redundancy analysis (Bray–Curtis) showing the differences in bacterial composition between AD and controls, and the association between bacterial composition and individual AD clinical variable (black vector). The *APOE* vector points toward the group of ε4 carrier samples. B, Differential abundance analysis showing the log‐fold change of bacterial ASVs with significantly different abundances in AD compared to controls. Red bars indicate higher abundances of ASVs in AD, and blue bars indicate lower abundances. Error bars denote one standard error. A standard error of zero indicates a structural zero (complete absence in one group of the variable). Aβ, amyloid beta; AD, Alzheimer's disease; *APOE*, apolipoprotein E; ASV, amplicon sequence variant; Dementia Years, number of years with dementia; Expired Age, deceased age; MMSE, Mini‐Mental State Examination score; Plaque Total, total number of plaques in the subject's brain; PMI, *post mortem* interval; Tangle Total, total number of tangles in the subject's brain

Given that multiple antimicrobial response pathways were downregulated in AD patients, we examined the composition of the microbiome present in the transverse colon samples. Beta diversity analyses revealed significant differences in bacterial composition between AD and control samples as assessed by the Bray–Curtis dissimilarity index (*P* = 0.021; Figure 4A). In addition, bacterial composition in AD samples was correlated with AD clinical features, such as longer dementia duration (*P* = 0.0013), higher plaque (*P* = 0.0099) and tangle burden (*P* = 0.0022), and lower MMSE scores (*P* = 0.017). PMI was also found significantly correlated with bacterial composition (*P* = 0.027), although this is not directly aligned with AD pathology. Alpha diversity analyses did not suggest any substantial differences in bacterial composition between AD and control samples (Figure S1 in supporting information).

More specifically, differential abundance analyses identified two ASVs, belonging to the *Christensenellaceae* family and *Desulfovibrio* genus, which were significantly more abundant in AD samples than in controls (Figure 4B). Meanwhile, three ASVs belonging to the *Streptococcus* genus, *Lachnospiraceae* family, and *Blautia* genus, were significantly less abundant in AD, in accordance with lower abundances of *Streptococcus*, *Lachnospiraceae*, and *Blautia* proteins identified in AD (Table S2 in supporting information). Moreover, significant associations between specific ASVs and clinical variables were observed (Figure S2 in supporting information). For example, a higher abundance of one *Lachnoclostridium* ASV correlated with longer dementia duration, while one *Enterobacteriaceae* ASV was associated with a shorter duration and less tangle burden. One *Blautia* ASV was linked to higher MMSE scores, whereas ASVs from the *Eggerthella* genus and the *Family XIII AD3011 group* genus (*Anaerovoracaceae* family) were negatively correlated with MMSE scores. *APOE* ε4 carrier status was associated with reduced abundances of several potential probiotics, including ASVs from *Blautia*, *Veillonella*, and *Bifidobacterium*.

Fungal composition was also investigated in AD and control samples (Figure S3 in supporting information). Although beta diversity analysis did not show any significant differences between the two groups, a few fungal ASVs, such as one *Candida tropicalis* ASV and one *Nakaseomyces* ASV, were differentially abundant. A subsequent analysis revealed that the *APOE* ε4 carrying status was the only clinical variable significantly associated with altered abundances of ASVs, including those from *Nakaseomyces* and *C. tropicalis* (Figure S4 in supporting information).

## 4 DISCUSSION

Our study provides evidence that alterations in the gut proteome and microbiome are associated with AD, offering insights into the gut–brain axis in neurodegeneration. By combining proteomic and genomic approaches, we identified distinct changes in gut protein expression and microbial composition in clinically confirmed AD patients compared to controls. These findings support the growing body of evidence linking systemic changes, particularly in the gut, to AD progression.^2^

### 4.1 Aβ in peripheral tissues

We confirmed the presence of Aβ species in the gut, with elevated Aβ42 levels detected in the transverse colon of AD patients. Aβ42, the species most strongly linked to AD pathology, raises important questions about the role of peripheral tissues in disease. Although prior studies have reported Aβ in peripheral tissues,^7^ its functional significance remains unclear.

The source of gut Aβ is also uncertain. It may originate in the brain and migrate peripherally, be produced locally in response to microbial activity, or reflect systemic amyloidogenesis in which peripheral tissues contribute to the overall amyloid burden.^39^

A key limitation in this study is that only Aβ42 was measured, without distinction between monomeric, oligomeric, or aggregated forms. Because monomeric Aβ42 is not inherently toxic, the pathogenic relevance of gut‐derived Aβ remains unresolved. Future work should include Aβ40 quantification, conformational and aggregation assays, and seeding models to test pathogenicity. Additionally, differences in post‐translational modifications and tissue‐specific Aβ species between brain and gut warrant further investigation to clarify systemic versus local contributions to AD.

### 4.2 Gut dysbiosis and its potential connection to AD pathology

We observed strong correlations between microbiome composition and clinical measures, including plaque burden and MMSE scores, suggesting that gut dysbiosis may reflect AD progression. These findings reinforce the therapeutic potential of microbiome modulation through diet, probiotics, or fecal microbiota transplantation.

In AD samples, *Christensenellaceae* and *Desulfovibrio* were significantly enriched, consistent with prior fecal microbiome studies.^40^, ^41^ *Christensenellaceae* has been linked to constipation and slow gut transit, both common in AD.^42^, ^43^ *Desulfovibrio* may contribute to pathology via excessive hydrogen sulfide production, which disrupts brain‐wave patterns and neurobehavior,^44^, ^45^ promotes α‐syn aggregation,^46^, ^47^ and generates magnetite nanoparticles capable of crossing the blood–brain barrier and facilitating α‐syn pathology.^48^, ^49^

Conversely, *Streptococcus*, *Blautia*, and *Lachnospiraceae* were reduced in AD. Lower *Streptococcus* glyceraldehyde‐3‐phosphate dehydrogenase suggests impaired carbohydrate metabolism and may reflect targeting by Aβ, which has antimicrobial activity.^50^ Decreases in *Blautia* and *Lachnospiraceae*, both butyrate producers,^51^, ^52^, ^53^ align with dementia‐associated microbiome changes.^14^, ^54^ Because butyrate supports epithelial integrity and suppresses neuroinflammation,^55^ their loss may further link gut dysbiosis to AD progression.

A higher abundance of *Lachnoclostridium* was linked to longer dementia duration, potentially via CNS inflammation.^56^ *Eggerthella*, associated with lower MMSE scores, is proinflammatory and implicated in bile acid dysregulation, possibly leading to cognitive decline.^57^ The role of *Family XIII AD3011 group* is less clear, though its positive association with AD and Aβ pathology aligns with our findings linking it to lower MMSE scores.^58^, ^59^ Interestingly, the *Enterobacteriaceae* ASV showed lower abundances in individuals with longer dementia duration and more tangles, despite previous reports linking *Enterobacteriaceae* enrichment to AD progression through mechanisms such as endotoxin production.^14^ These contrasting observations highlight the complexity of microbial associations with dementia, suggesting that differences between colon tissue–based and fecal microbiome studies merit additional study.

Fungal alterations of *C. tropicalis* and *Nakaseomyces* warrant further investigation. The ability of these fungi to influence immune responses or migrate to the CNS highlights their potential involvement in AD pathogenesis.^60^, ^61^

### 4.3 Biological pathways dysregulation in AD

We observed significant downregulation of antimicrobial humoral and oxidative stress response pathways in AD colon. Reduced antimicrobial defense, particularly against Gram‐positive bacteria and fungi, may compromise gut immunity and contribute to the altered microbiome. Although our analysis did not show Gram‐positive enrichment, the higher abundance of pathogenic *C. tropicalis* in AD may reflect weakened antimicrobial responses. The decreased oxidative stress response further suggests reduced resilience against oxidative damage, consistent with brain studies linking oxidative stress to neurodegeneration.^62^ Dysbiosis and oxidative stress may therefore interact to fuel systemic inflammation and AD progression.

In contrast, metabolic pathways including carboxylic and fatty acid catabolism were upregulated.^63^ Notably, insulin receptor signaling was also elevated. Although brain insulin signaling is typically reduced in AD, peripheral tissues can show early hyperactivation as a compensatory mechanism under systemic insulin resistance (e.g., glucagon‐like peptide‐1 [GLP‐1]–mediated upregulation of insulin receptor beta, insulin receptor substrate 1, and glucose transporter type 4 in adipose tissue).^64^, ^65^, ^66^ The gastrointestinal tract, via enteroendocrine mediators such as GLP‐1, contributes to systemic insulin sensitivity through the gut–brain–pancreas axis.^65^, ^67^ Thus, increased insulin signaling in the colon may reflect adaptive responses to metabolic stress rather than contradicting central insulin resistance.

Finally, pathways related to transition metal ion homeostasis, including zinc, were altered in AD. Dysregulated metal balance can promote oxidative stress and protein misfolding, central to AD pathology.^68^

Together, these findings highlight a dynamic interplay among immunity, metabolism, and microbiome alterations in the gut, supporting its active role in AD pathogenesis.

### 4.4 Synaptic dysfunction in AD

Synaptic dysfunction is an early hallmark of AD in the brain,^69^, ^70^ and our findings suggest that similar alterations occur in the ENS. In AD colon tissues, several key presynaptic and postsynaptic proteins were significantly reduced. SYP, a vesicle glycoprotein essential for synaptic trafficking and neurotransmitter release, and UNC13B, which facilitates vesicle priming at excitatory synapses,^37^, ^38^ were markedly downregulated (5.3‐ and 1.6‐fold, respectively). Such changes may impair synaptic transmission and gut neural circuit function. Additional reductions were seen in DOCK1, a regulator of axon guidance; GPHN, a scaffolding protein clustering inhibitory receptors; and HTT and INA, which maintain cytoskeletal integrity and neuronal survival.^71^, ^72^, ^73^, ^74^, ^75^, ^76^ Collectively, these findings point to a weakening of both excitatory and inhibitory synaptic networks in the ENS.

In contrast, several proteins were upregulated. FXYD6, a neuronal modulator of Na⁺/K⁺‐ATPase activity that regulates excitability and signaling,^77^, ^78^ and PRKCE, a kinase involved in plasticity, learning, and stress responses,^79^, ^80^ showed significant increases. These changes may represent adaptive remodeling under pathological stress.

Together, the downregulation of core presynaptic and scaffolding proteins alongside the upregulation of stress‐responsive regulators aligns with previous reports of ENS dysfunction in AD, including neuronal loss, cholinergic deficits, impaired motility, and amyloid/tau accumulation.^81^, ^82^, ^83^ These results support the view that the ENS mirrors CNS pathology in AD, undergoing both degenerative and compensatory changes that may contribute to gastrointestinal and autonomic disturbances.

### 4.5 Complement system dysregulation in AD

We identified a general downregulation of complement‐related proteins in AD, an important finding given the complement system's role in immune surveillance, inflammation, and tissue homeostasis.^84^, ^85^ Downregulated proteins included C2, C3, C4A, C4B, C5, C6, and C7, with C5, C6, and CFB showing modest significance (adjusted *P*s = 0.050–0.051) and CFI, a key regulator, showing the most significant reduction (adjusted *P* = 0.013). This pattern suggests impaired complement‐mediated immune defense and clearance functions, potentially increasing susceptibility to infection, disrupting gut immune balance, and hindering removal of apoptotic cells or aggregates, processes that may exacerbate inflammation and neurodegeneration.

Notably, complement suppression in the transverse colon contrasts with the well‐established hyperactivation seen in AD brains.^86^ One explanation is that reduced gut complement activity compromises microbial clearance, promoting dysbiosis and intestinal permeability. This could allow bacterial products to enter circulation, triggering peripheral immune activation and microglial priming, thereby fueling brain complement overactivation.^87^, ^88^ Such a model aligns with evidence for bidirectional immune crosstalk along the gut–brain axis, linking intestinal dysfunction to central neuroinflammation in AD.^89^

Despite the overall suppression, some complement‐related proteins (e.g., CD55 and C9) were upregulated, though not significantly. This mixed profile may suggest a regulatory imbalance rather than uniform loss of complement activity, reflecting the dual protective and pathogenic roles of complement.

We note that direct measures of intestinal barrier integrity (e.g., tight junction markers, histology, and permeability assays) were not performed. Thus, the proposed role of gut barrier dysfunction in AD pathogenesis remains hypothetical in this context, and future studies incorporating functional and molecular assays of intestinal barrier status will be necessary to test this model.

### 4.6 Limitations and future directions

By integrating proteomic and microbiome analyses, this study highlights the gut as both a potential contributor to and therapeutic target for AD pathology. However, its cross‐sectional design prevents causal inference, and key confounders (e.g., diet, medication use, and systemic metabolic status) were not controlled. Moreover, all participants were of European ancestry, which may limit generalizability. Longitudinal and interventional studies will be required to clarify the temporal dynamics and causal relevance of gut‐derived molecular changes in AD.

Moreover, we observed proteomic signatures consistent with enteric synaptic dysfunction but did not assess fecal samples, leaving unexplored the potential for non‐invasive biomarkers of neuronal protein changes. Future work should determine how well fecal microbial profiles reflect gut mucosa and whether combined microbiome–proteome signatures can identify diagnostic or prognostic markers. Such approaches could ultimately support the development of non‐invasive screening tools and gut‐targeted therapies for AD.

## CONFLICT OF INTEREST STATEMENT

The authors declare no conflicts of interest. Author disclosures are available in the supporting information.

## CONSENT STATEMENT

All Banner participants were volunteers in the AZSAND, a longitudinal clinicopathological study of aging, cognition, and movement in the elderly since 1996 in Sun City, Arizona. Autopsies are performed by the Banner Sun Health Research Institute Brain and Body Donation Program (BBDP; www.brainandbodydonationprogram.org). All subjects sign institutional review board‐approved informed consents allowing both clinical assessments during life and several options for brain and/or bodily organ donation after death.

## Supporting information



Supporting information

Supporting information
