# Supplementary material for: Gut proteome and microbiome alterations: Analysis of transverse colon samples from pathologically confirmed Alzheimer's disease patients
Source: Alzheimers Dement. 2026 Jan 13;22(1):e71021. doi: 10.1002/alz.71021 (PMC12796839; doi:10.1002/alz.71021)
Supplement: Supplementary file 1 — Supporting information [file ALZ-22-e71021-s001.docx]

**Supplementary Table 1** Autopsy subjects and metadata.

| **CaseID** | **Race** | **Gender** | **Expired Age** | **PMI** | **MMSE** | ***APOE*** | **Plaque**  **Total** | **Tangle Total** | **Braak Score** | **Neurological**  **Diagnosis** | **Dementia**  **Years** | **Aβ42 pg/ml** |
| --- | --- | --- | --- | --- | --- | --- | --- | --- | --- | --- | --- | --- |
| 05-52 | 1 | 2 | 70 | 2.7 | 5 | 3/3 | 15 | 15 | VI | AD | 4 | 99 |
| 07-54 | 1 | 2 | 82 | 4 | 4 | 3/3 | 15 | 15 | VI | AD | 11 | 68 |
| 08-37 | 1 | 1 | 87 | 2.5 | 13 | 2/3 | 15 | 14.5 | VI | AD | 6 | 90 |
| 08-45 | 1 | 1 | 78 | 2.3 | 7 | 3/4 | 14 | 15 | VI | AD | 5 | 46 |
| 09-39 | 1 | 1 | 82 | 3 | 2 | 3/4 | 15 | 15 | VI | AD | 12 | 91 |
| 09-42 | 1 | 1 | 85 | 3 | 23 | 3/3 | 15 | 15 | VI | AD | 7 | 98 |
| 09-61 | 1 | 1 | 91 | 3.3 | 0 | 4/4 | 15 | 15 | VI | AD | 21 | 53 |
| 11-76 | 1 | 1 | 75 | 5 | 6 | 3/4 | 13.5 | 15 | VI | AD | 10 | 47 |
| 11-38 | 1 | 1 | 64 | 2.3 | 1 | 3/3 | 14.5 | 15 | VI | AD | 9 | 65 |
| 12-27 | 1 | 2 | 89 | 2.7 | 11 | 3/3 | 15 | 15 | VI | AD | 2 | 95 |
| 12-49 | 1 | 1 | 63 | 5.2 | 14 | 3/4 | 14.5 | 15 | VI | AD | 10 | 83 |
| 13-10 | 1 | 1 | 74 | 2.6 | 14 | 3/4 | 14 | 14 | VI | AD | 4 | 63 |
| 13-31 | 1 | 1 | 85 | 3.6 | 16 | 3/4 | 13.5 | 13 | VI | AD | 14 | 68 |
| 13-42 | 1 | 1 | 80 | 3 | 27 | 4/4 | 14 | 12 | VI | AD | 20 | 48 |
| 13-66 | 1 | 1 | 75 | 3.8 | 3 | 3/4 | 15 | 15 | V | AD | 7 | 28 |
| 13-75 | 1 | 1 | 77 | 3.6 | 10 | 3/4 | 15 | 15 | VI | AD | 12 | 61 |
| 14-06 | 1 | 1 | 85 | 3.5 | 17 | 3/4 | 14 | 14.5 | V | AD | 11 | 55 |
| 14-22 | 1 | 1 | 88 | 3.9 | 14 | 3/4 | 13.5 | 15 | V | AD | 3 | 14 |
| 14-48 | 1 | 1 | 73 | 4.8 | 7 | 3/4 | 14.5 | 15 | VI | AD | 11 | 57 |
| 15-27 | 1 | 2 | 84 | 3.3 | 24 | 3/3 | 15 | 11 | VI | AD | 1 | 57 |
| 16-18 | 1 | 1 | 91 | 3 | 8 | 2/4 | 14.5 | 14 | VI | AD | 14 | 98 |
| 17-11 | 1 | 2 | 89 | 2.4 | 3 | 3/4 | 9 | 8.5 | VI | AD | 10 | 70 |
| 17-58 | 1 | 2 | 93 | 2.2 | 13 | 3/3 | 15 | 11 | V | AD | 7 | 52 |
| 17-60 | 1 | 2 | 82 | 4 | 19 | 3/3 | 15 | 14.5 | VI | AD | 15 | 66 |
| 18-74 | 1 | 1 | 89 | 2.2 | 24 | 3/3 | 14.5 | 9.5 | V | AD | 1 | 60 |
| 18-79 | 1 | 2 | 94 | 3.5 | 22 | 3/3 | 12 | 15 | V | AD | 12 | 74 |
| 06-15 | 1 | 2 | 87 | 2.5 | 26 | 2/3 | 4.5 | 4.25 | III | Control | N/A | 65 |
| 06-19 | 1 | 1 | 84 | 4.3 | 30 | 2/3 | 7 | 1.25 | II | Control | N/A | 13 |
| 06-21 | 1 | 1 | 73 | 2.5 | 30 | 2/3 | 5.5 | 3 | II | Control | N/A | 12 |
| 07-11 | 1 | 2 | 75 | 2.8 | 29 | 3/4 | 0 | 5 | III | Control | N/A | 12 |
| 07-37 | 1 | 1 | 89 | 5.5 | 30 | 3/3 | 0 | 2 | II | Control | N/A | 18 |
| 08-40 | 1 | 1 | 76 | 2.3 | 29 | 3/4 | 5.5 | 0 | I | Control | N/A | 55 |
| 08-55 | 1 | 1 | 71 | 3 | 26 | 3/3 | 0 | 0 | I | Control | N/A | 18 |
| 08-64 | 1 | 2 | 68 | 2.6 | 29 | 3/3 | 0 | 3.5 | III | Control | N/A | 23 |
| 08-88 | 1 | 1 | 65 | 3.5 | 29 | 3/3 | 0 | 1 | I | Control | N/A | 10 |
| 08-90 | 1 | 1 | 81 | 2.3 | 29 | 3/3 | 0 | 4.5 | III | Control | N/A | 54 |
| 09-20 | 1 | 2 | 99 | 3.5 | 29 | 3/3 | 0 | 3.5 | III | Control | N/A | 16 |
| 09-57 | 1 | 1 | 80 | 3.5 | 28 | 2/3 | 4.5 | 5 | III | Control | N/A | 13 |
| 10-22 | 1 | 2 | 59 | 3.2 | 29 | 3/3 | 1 | 0.5 | I | Control | N/A | 16 |
| 10-26 | 1 | 2 | 95 | 2.5 | 28 | 3/3 | 0 | 6.5 | III | Control | N/A | 24 |
| 10-39 | 1 | 1 | 93 | 3 | 30 | 3/3 | 0 | 1 | I | Control | N/A | 24 |
| 10-63 | 1 | 1 | 79 | 3 | 29 | 3/3 | 0 | 2.5 | II | Control | N/A | 27 |
| 10-70 | 1 | 1 | 74 | 3.3 | 30 | 2/3 | 0 | 2 | I | Control | N/A | 21 |
| 11-07 | 1 | 2 | 93 | 3.2 | 25 | 3/3 | 12 | 7.5 | IV | Control | N/A | 21 |
| 12-21 | 1 | 2 | 88 | 1.3 | 29 | 3/4 | 14 | 3.5 | II | Control | N/A | 13 |
| 13-49 | 1 | 2 | 75 | 2.5 | 28 | 3/3 | 0 | 2.5 | II | Control | N/A | 17 |
| 14-30 | 1 | 1 | 87 | 3.6 | 28 | 2/3 | 0 | 4.5 | III | Control | N/A | 9.4 |
| 15-08 | 1 | 1 | 78 | 3.3 | 28 | 3/3 | 0 | 3.5 | II | Control | N/A | 17 |
| 15-46 | 1 | 2 | 82 | 2.1 | 29 | 2/3 | 1 | 1.5 | I | Control | N/A | 22 |
| 15-78 | 1 | 2 | 93 | 2.9 | 29 | 3/3 | 13.5 | 4 | III | Control | N/A | 48 |
| 16-11 | 1 | 1 | 90 | 5.4 | 22 | 3/3 | 0 | 6 | III | Control | N/A | 21 |
| 16-32 | 1 | 1 | 79 | 4.3 | 28 | 2/3 | 0 | 1 | II | Control | N/A | 37 |
| 18-17 | 1 | 2 | 87 | 3.1 | 28 | 3/4 | 0 | 4.5 | III | Control | N/A | 24 |
| 18-78 | 1 | 1 | 71 | 3.5 | 27 | 3/3 | 0 | 4 | III | Control | N/A | 18 |

*Note*: Demographic information includes unique CaseID, Race (numerically encoded 1=Caucasian), Gender (numerically encoded 1= Male, 2= Female), and Expired Age (age at the time of death). Clinical data provided includes PMI (post-mortem interval, defined as the time in hours between death and autopsy), and the most recent MMSE (Mini-Mental State Examination) score prior to death. The table also specifies the subject's *APOE* (apolipoprotein E) genotype (e.g., 3/3, 2/3). Neuropathological measures include Plaque Total (the total count of plaques) and Tangle Total (the total count of neurofibrillary tangles), along with Braak Scores that indicate the stage of Alzheimer’s Disease (AD) pathology. Additional columns document the subject’s Neurological Diagnosis (AD or Control), Dementia Years (the duration of dementia in years), and Aβ42 (amyloid β42 levels in pg/mL). This dataset provides a foundation for analyzing correlations between neuropathological findings, clinical variables, and demographic characteristics.

**Supplementary Table 2** Bacterial proteins exhibiting significantly different abundances in the transverse colon of Alzheimer's Disease (AD) patients compared to controls.

| **Protein** | **Biological process** | **Gene symbol** | **Log fold change in AD vs controls** | **Adjusted *P*-value** |
| --- | --- | --- | --- | --- |
| **Streptococcus** |  |  |  |  |
| glyceraldehyde-3-phosphate dehydrogenase, type I [*Streptococcus infantarius* subsp. *infantarius* ATCC BAA-102] | other metabolic processes | gapA | -0.99 | .004 |
| glyceraldehyde-3-phosphate dehydrogenase [*Streptococcus anginosus* 1_2_62CV] | other metabolic processes | gap; gapA | -0.99 | .004 |
| glyceraldehyde-3-phosphate dehydrogenase [*Streptococcus* sp. HPH0090] | other metabolic processes |  | -0.99 | .004 |
|  |  |  |  |  |
| **Lachnospiraceae** |  |  |  |  |
| elongation factor Tu [*Lachnospiraceae bacterium* 9_1_43BFAA] | protein metabolism;other metabolic processes | tuf | -0.76 | .058 |
| translation elongation factor Tu [*Lachnospiraceae* [*Clostridium*] *hathewayi* DSM 13479] | protein metabolism;other metabolic processes | tuf | -0.76 | .058 |
| elongation factor Tu [*Lachnospiraceae* [*Clostridium*] *hathewayi* WAL-18680] | protein metabolism;other metabolic processes | tuf | -0.76 | .058 |
| elongation factor Tu [*Lachnospiraceae bacterium* 2_1_46FAA] | protein metabolism;other metabolic processes | tuf | -0.76 | .058 |
| translation elongation factor 1A (EF-1A/EF-Tu) [*Lachnospiraceae* [*Clostridium*] cf. *saccharolyticum* K10] | protein metabolism;other metabolic processes | tuf | -0.76 | .058 |
| translation elongation factor Tu [*Lachnospiraceae bacterium* 5_1_63FAA] | protein metabolism;other metabolic processes | tuf | -0.76 | .058 |
| elongation factor Tu [*Lachnospiraceae* [*Clostridium*] *clostridioforme* 2_1_49FAA] | protein metabolism;other metabolic processes | tuf | -0.76 | .058 |
| translation elongation factor Tu [*Lachnospiraceae* [*Clostridium*] *hylemonae* DSM 15053] | protein metabolism;other metabolic processes | tuf | -0.76 | .058 |
| elongation factor Tu [*Lachnospiraceae* [*Clostridium*] *citroniae* WAL-17108] | protein metabolism;other metabolic processes | tuf | -0.76 | .058 |
|  |  |  |  |  |
| **Blautia** |  |  |  |  |
| translation elongation factor Tu [*Blautia hydrogenotrophica* DSM 10507] | protein metabolism;other metabolic processes | tuf | -0.76 | .058 |
| translation elongation factor Tu [*Blautia hansenii* DSM 20583] | protein metabolism;other metabolic processes | tuf | -0.76 | .058 |

*Note*: Proteins are grouped by their taxonomic families, including *Streptococcus*, *Lachnospiraceae*, and *Blautia*, reflecting taxa with significant changes in abundance. Negative log fold change values represent downregulation in AD compared to controls, while positive values represent upregulation. Proteins with significant changes (adjusted *P*s < .05) highlight potential biological processes altered in AD pathology. Proteins from *Streptococcus*, such as glyceraldehyde-3-phosphate dehydrogenase (gapA), were significantly downregulated in AD samples. *Lachnospiraceae* proteins, predominantly elongation factor Tu (tuf), also showed consistent downregulation, indicating reduced protein metabolism and other metabolic processes. Similarly, *Blautia* proteins, including translation elongation factor Tu, exhibited decreased expression in AD samples.


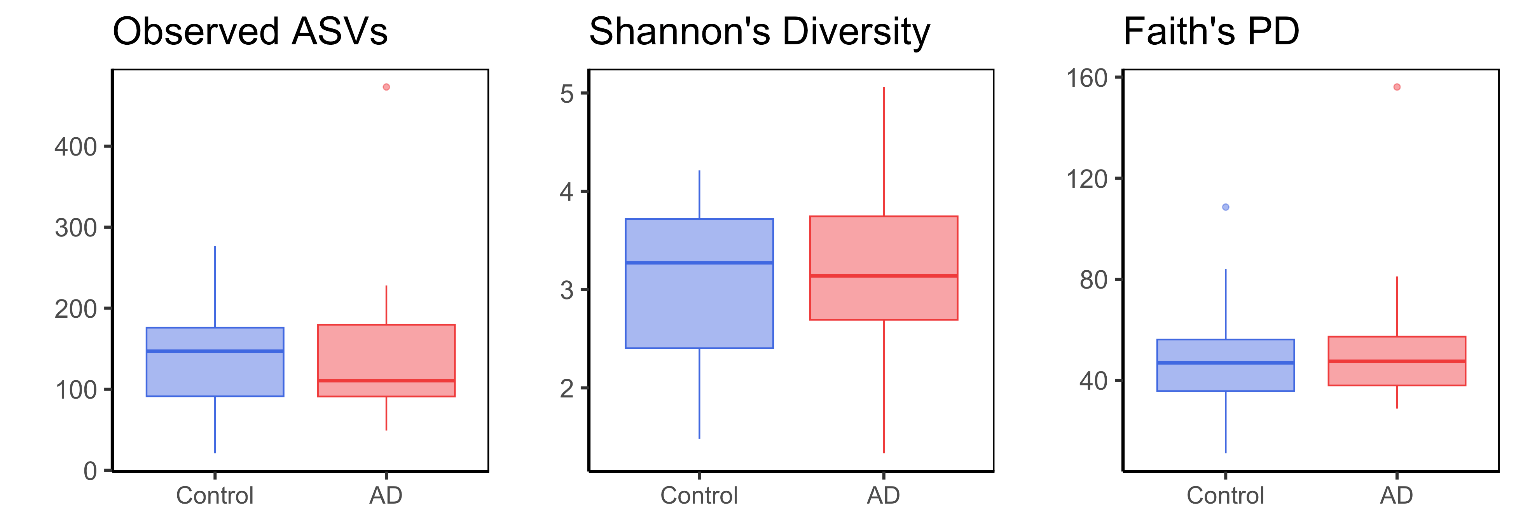


**Supplementary Figure 1** Comparison of bacterial alpha diversity metrics between Alzheimer’s Disease (AD) patients and controls. No significant differences could be observed (*P*s > .05). Observed ASVs: Observed Amplicon Sequence Variants; Faith’s PD: Faith’s Phylogenetic Diversity.

**
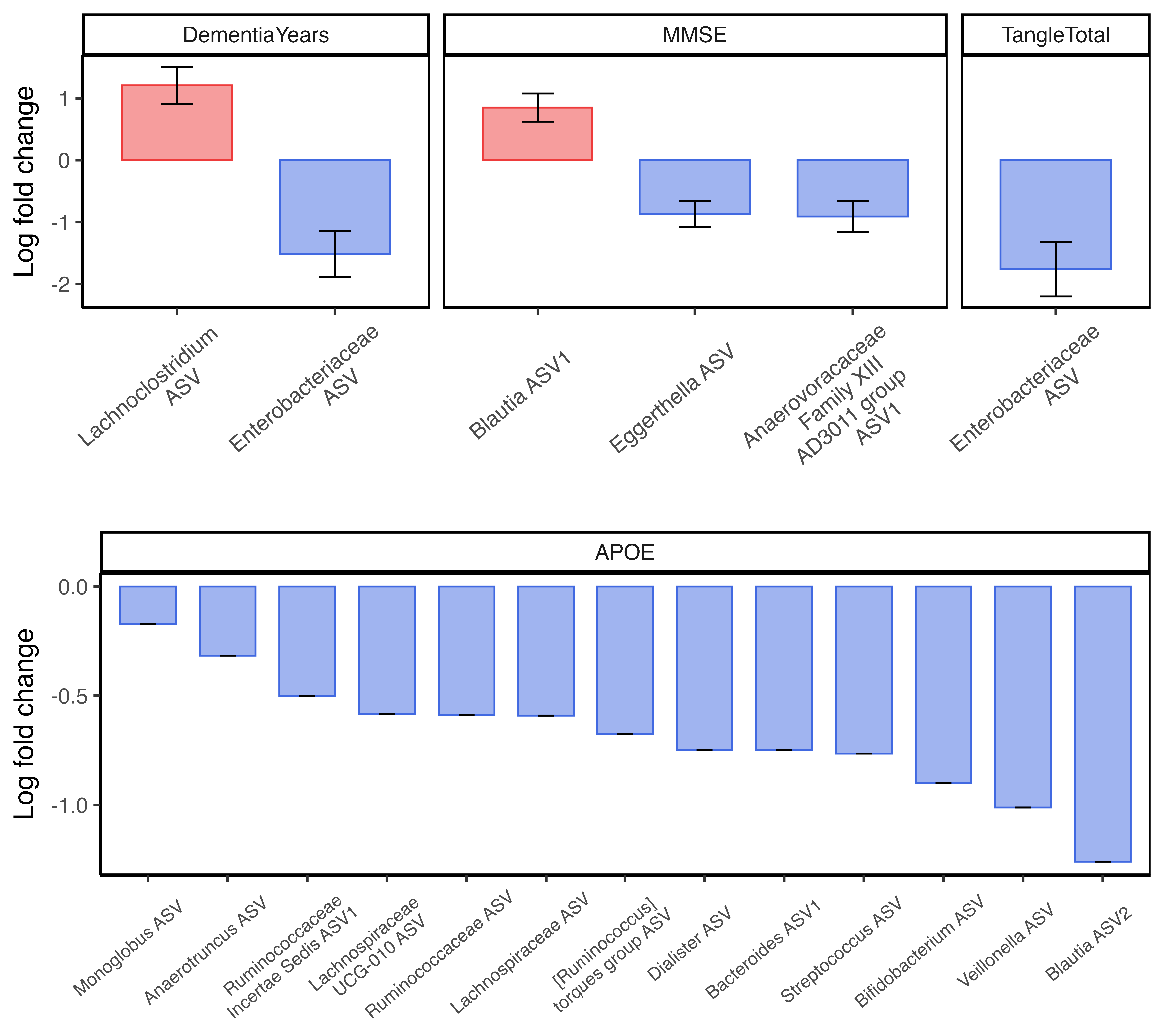
**

**Supplementary Figure 2** Differential abundance of bacterial amplicon sequence variants (ASVs) across key clinical variables. Red and blue bars indicate positive and negative correlations between the ASV abundance and variable, respectively. Error bars denote one standard error. A standard error of zero indicates a structural zero (complete absence in one group of the variable). DementiaYears: number of years the subject had suffered from dementia; MMSE: Mini-Mental Status Exam score; TangleTotal: total number of tangles in the subject’s brain; APOE: apolipoprotein E4 carrier status, with blue bars indicating lower abundances of ASVs in samples carrying at least one copy of E4.

**
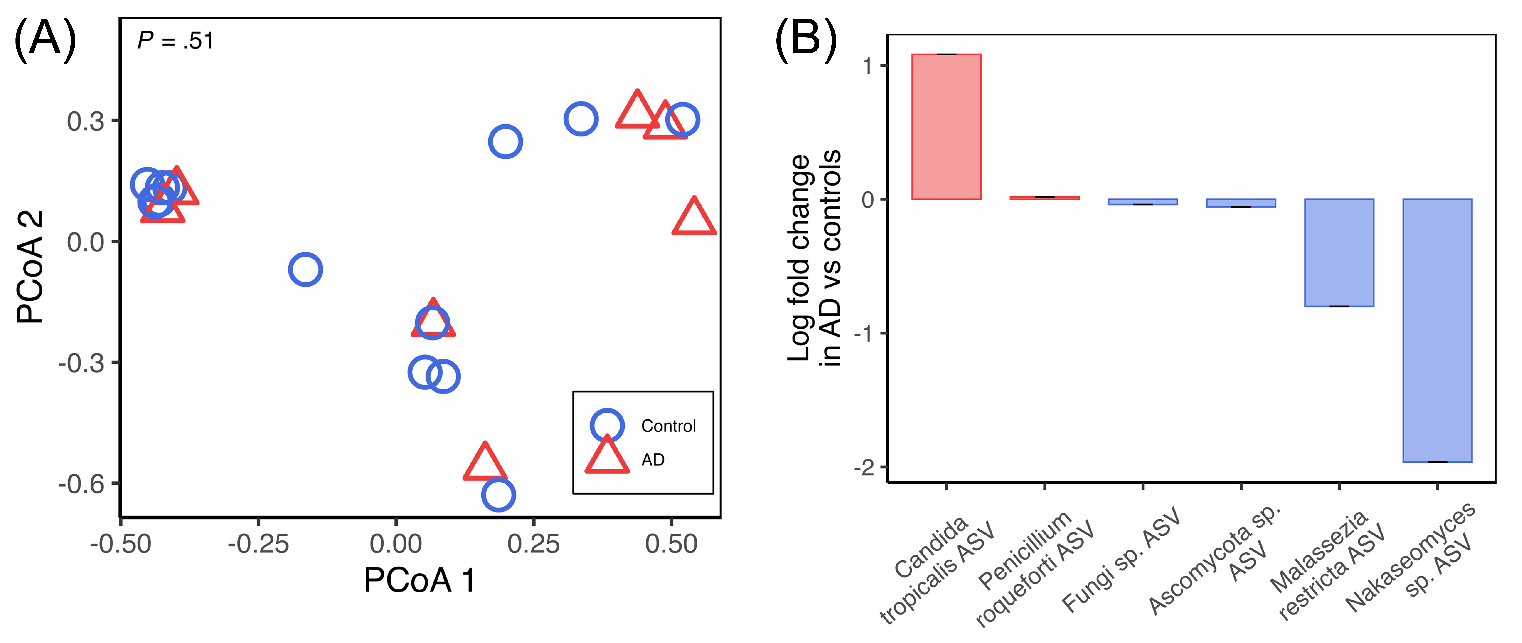
**

**Supplementary Figure 3** Comparison of fungal composition in the transverse colon between Alzheimer’s Disease (AD) patients and controls. (A) Bray–Curtis dissimilarity showing the differences in fungal composition between AD and controls. No significant differences could be observed. (B) Differential abundance analysis showing the log fold change of fungal amplicon sequence variants (ASVs) with significantly different abundances in AD compared to controls. Red bars indicate higher abundances of ASVs in AD, and blue bars indicate lower abundances. Error bars denote one standard error. A standard error of zero indicates a structural zero (complete absence in one group of the variable).

**
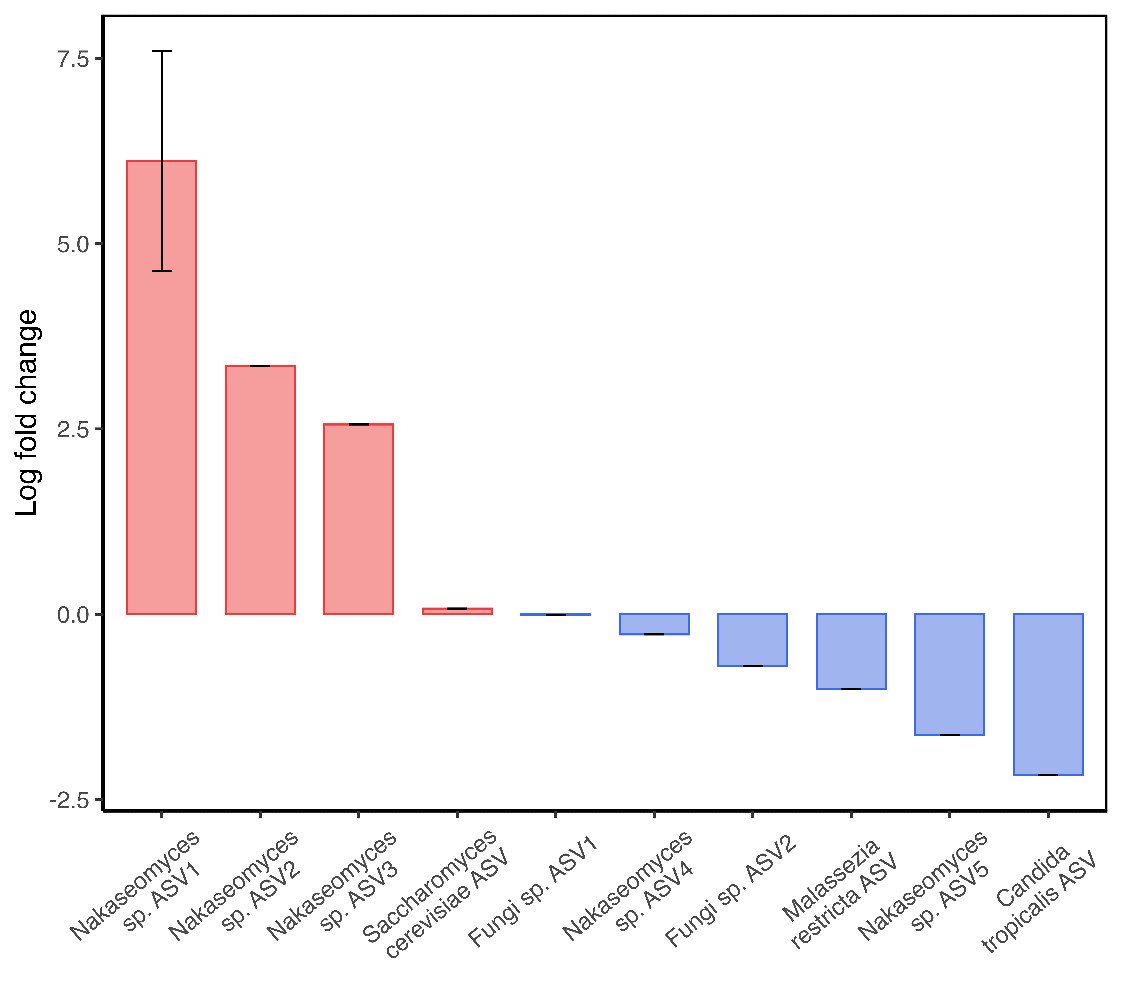
**

**Supplementary Figure 4** Differential abundance of fungal amplicon sequence variants (ASVs) in apolipoprotein E4 carriers compared to non-carriers. Red and blue bars indicate higher and lower abundances of ASVs in carriers and non-carriers, respectively. Error bars denote one standard error. A standard error of zero indicates a structural zero (complete absence in one group of the variable).
